# Garlic burns are not burns

**DOI:** 10.51866/lte.955

**Published:** 2025-07-14

**Authors:** Jazlan Jamaluddin, Siti Nuradliah Jamil, Siti Khamsiah Abd Shukor

**Affiliations:** 1 MD, MMed (Fam Med), Department of Primary Care Medicine, Faculty of Medicine, Universiti Malaya, Kuala Lumpur, Malaysia. Email: jazlan@um.edu.my; 2 MD, MMed (Fam Med), Klinik Kesihatan Pasir Gudang, JKR 5376, Jalan 8/1 Perjiranan 8, Pasir Gudang, 81700, Johor Bahru, Johor, Malaysia.; 3 MD, MMed (Fam Med), AOI Wound Care Trainee, Klinik Kesihatan Batu Arang, Pejabat Kesihatan Daerah Gombak, Gombak, Selangor, Malaysia.

**Keywords:** Garlic, Contact dermatitis, Burns, Irritant dermatitis

**Dear editor,**

We read with great interest the recent case report by Amal Lina et al.^1^ While we commend the authors for highlighting the potential risks of unregulated traditional remedies, we believe the terminology employed - ‘garlic burn’ - warrants scientific reconsideration. Notably, while the authors initially identified the lesion as irritant contact dermatitis (ICD) in the case presentation, the discussion section appears to revert to the more ambiguous and potentially misleading label of ‘burn’.^2^ This distinction is not merely semantic but has important implications for diagnosis, management, patient education and public health campaign.

The term ‘burn’ refers to tissue injury resulting from exposure to heat, caustic chemicals, electricity or radiation. True chemical burns, such as those induced by strong acids or alkalis, lead to immediate coagulative necrosis and widespread protein denaturation. In contrast, garlic (Allium sativum) lacks the corrosive profile of classical chemical agents. Its cutaneous effects result from biochemical irritation rather than thermal or caustic mechanisms. When garlic is crushed, the enzyme alliinase converts alliin to allicin - a highly reactive sulphur-containing compound capable of forming disulfide bonds with thiol groups in epidermal proteins. This biochemical interaction disrupts keratinocyte membranes, induces oxidative stress and initiates a local inflammatory cascade.^3,4^ The resulting skin damage is dose-dependent, non-immunologic and exacerbated by occlusion, as in the reported case where garlic was applied under a bandage overnight. This profile aligns closely with the pathophysiology of ICD, which is characterised by local cytotoxic reactions to external agents, particularly when barrier function is compromised or prolonged exposure occurs.

This assertion is not only theoretical but also well-supported by clinical and histological evidence. Numerous reports describe garlic-induced skin lesions exhibiting classic features of ICD, including spongiosis, necrotic keratinocytes and superficial dermal inflammation.^5,6^ Moreover, these cases often yield negative patch test results for diallyl disulfide - the primary allergen in garlic - further excluding allergic contact dermatitis as a diagnosis. In the paediatric case by Amal Lina et al., the delayed onset of blistering, absence of systemic toxicity and full resolution with conservative management are more consistent with irritant dermatitis than with a true burn.^1^ Similarly, a systematic review found that the majority of garlic-related injuries corresponded to partial-thickness ICD, particularly when fresh garlic was applied for prolonged periods under occlusion.^7^ Although these lesions may mimic second-degree burns morphologically - manifesting as erythema, vesicle formation and epidermal sloughing - the underlying pathogenesis is distinctly irritant in nature.

The widespread use of the term ‘garlic burn’ in scientific literature, clinical documentation and media coverage risks perpetuating misconceptions about both the cause and appropriate management of such injuries. This mislabelling can lead to suboptimal treatment approaches, including overhydration, unnecessary surgical interventions or delayed initiation of anti-inflammatory therapy. In contrast, correctly identifying these injuries as ICD guides clinicians towards targeted interventions, such as the application of emollients or potent topical corticosteroids and avoidance of further exposure.

Beyond clinical accuracy, this reclassification holds value in public health communication. The label ‘burn’ may downplay the role of the compound’s biological activity and instead evoke images of accidental scalding or thermal injury. Educating the public about the mechanism of irritant dermatitis - particularly in the context of herbal self-medication - can help improve patient understanding, promote safer practices and discourage reliance on unverified folk remedies, especially in paediatric populations where the skin barrier is more vulnerable.

In conclusion, we strongly advocate for a terminological shift from ‘garlic burn’ to ‘garlic- induced ICD’. This nomenclature better reflects the underlying pathophysiology, aligns with histopathological evidence and enhances the precision of clinical discourse. As the term ‘burn’ has no suitable alternative in medical taxonomy, it should not be used interchangeably with garlic-related ICD. In an era where traditional medicine increasingly intersects with evidence-based care, clarity in language is not a luxury but a necessity.
